# Diurnal changes in seawater carbonate chemistry speciation at increasing atmospheric carbon dioxide

**DOI:** 10.1007/s00227-012-1965-y

**Published:** 2012-06-13

**Authors:** K. G. Schulz, U. Riebesell

**Affiliations:** Helmholtz Centre for Ocean Research (GEOMAR), Düsternbrooker Weg 20, 24105 Kiel, Germany

## Abstract

Natural variability in seawater pH and associated carbonate chemistry parameters is in part driven by biological activities such as photosynthesis and respiration. The amplitude of these variations is expected to increase with increasing seawater carbon dioxide (CO_2_) concentrations in the future, because of simultaneously decreasing buffer capacity. Here, we address this experimentally during a diurnal cycle in a mesocosm CO_2_ perturbation study. We show that for about the same amount of dissolved inorganic carbon (DIC) utilized in net community production diel variability in proton (H^+^) and CO_2_ concentrations was almost three times higher at CO_2_ levels of about 675 ± 65 in comparison with levels of 310 ± 30 μatm. With a simple model, adequately simulating our measurements, we visualize carbonate chemistry variability expected for different oceanic regions with relatively low or high net community production. Since enhanced diurnal variability in CO_2_ and proton concentration may require stronger cellular regulation in phytoplankton to maintain respective gradients, the ability to adjust may differ between communities adapted to low in comparison with high natural variability.

## Introduction

There is a considerable natural variability in seawater carbonate chemistry speciation, namely carbon dioxide (CO_2_), bicarbonate (HCO_3_^−^), carbonate ion (CO_3_^2−^) and proton (H^+^) concentration, as well as pH and calcium carbonate saturation state $(\Upomega)$. This is mainly caused by changes in temperature and biological activities such as photosynthesis, respiration, nutrient utilization, remineralization and calcium carbonate precipitation and dissolution. Variability is on inter-annual, seasonal and diurnal time scales.

For instance, photosynthesis and respiration have been reported to drive diurnal variations in pH of up to one unit in eutrophic lakes [compare Maberly (1996)]. In seawater, diurnal fluctuations in pH are usually considerably smaller, ranging from 0.1 units in spring in the Bay of Calvi in the Mediterranean (Frankignoulle and Bouquegneau 1990) to 0.15 in autumn in the Bay of Bengal in the Indian Ocean (Subramanian and Mahadevan 1999) and up to 0.5 in a Kelp forest close to the Kerguelen Archipelago in the Southern Ocean in austral summer (Delille et al. 2009). In coral reefs, calcium carbonate precipitation and dissolution are impacting carbonate chemistry speciation apart from photosynthesis and respiration, and measured pH changes range from 0.16 to 0.78 units [Ohde and van Woesik 1999; Frankignoulle et al. 1996. Tides, however, may also contribute to diurnal changes considerably (Manzello 2010)].

Similarly, seasonal variations differ from region to region with highest pH variability in low-buffered eutrophic systems such as lakes or the Baltic Sea with up to 3.2 and 0.7 pH units, respectively (Maberly 1996; Thomsen et al. 2010). Lowest seasonal variability is found in well-buffered oligotrophic open ocean waters with an average of about 0.022 at HOT, the Hawaii Ocean Time Series, and 0.055 pH units at ESTOC, the European Station for Time Series in the Ocean [adapted from Dore et al. (2009) and González-Dávila and Santana-Casiano (2011), respectively].

On top of the natural carbonate chemistry variability, the ongoing release of anthropogenic CO_2_ is shifting speciation toward higher concentrations of CO_2_, HCO_3_^−^ and H^+^, reducing pH, CO_3_^2−^ concentrations and calcium carbonate saturation state. Reduced CO_3_^2−^ ion concentrations lower the buffer capacity of seawater, thus considerably increasing natural variability (Frankignoulle 1994).

Here, we assess this phenomenon experimentally by reporting on diurnal variations in seawater carbonate chemistry speciation during a phytoplankton bloom event in five mesocosms manipulated for different seawater CO_2_ levels, ranging initially from about 370 μatm to 1250 μatm. These levels are representative for current day conditions and, according to different emission scenarios (Farquhar et al. 2001), the upper limits expected at the end of this century, respectively. Furthermore, a modeling exercise demonstrates that changes in the magnitude of natural variability are expected to differ between oceanic regions.

## Materials and Methods

### Experimental setup

In May 2009, six off-shore mesocosms were deployed by the research vessel R/V ALKOR at Boknis Eck in the Kiel Bight in the western part of the Baltic Sea (∼54.53° N, ∼10.03° E). The Kiel Off-Shore Mesocosms for future Ocean Simulations (KOSMOS) were moored in clusters of three in about 1 nautical mile distance to the shoreline. For a detailed description of KOSMOS construction and operation, see Riebesell et al. [in prep.]. Briefly, 13.5-m-long and 2-m-diameter thermoplastic polyurethane bags were attached to 7-meter-long floating structures, rising about 1.5 m out of the water, and unfolded to about 12 m depth. For this procedure, flaps at the bottom of the bags were opened to ensure thorough filling. Then, the upper part of the bags was pulled about 1 m underneath the water surface to allow for seawater exchange at both ends, minimizing any initial differences between the enclosed water masses of the six mesocosm bags. After about two days, differences in profiles of salinity, temperature, turbidity, chlorophyll *a* (Chl *a*) and pH were found to be negligible as measured by multiple CTD casts (see "CTD operation" section for details). A team of divers then closed the flaps at the bottom of the bags, while a second group quickly retrieved the upper part of the bags from 1 m depth and secured it 1.5 m above the water surface to the floating structures, thereby enclosing seawater (in theory 37.7 m^3^, but see below) with very similar starting conditions in all six bags.

The seawater carbonate system in the six bags was manipulated gradually over three days by injections of certain amounts of CO_2_-enriched filtered seawater (20 μm). This was achieved by lowering a dispersal device to 12 m depth and pulling it up again to the surface for several times. Pumping of the CO_2_-enriched seawater through the dispersal device then evenly distributed the CO_2_ addition between 0 and 12 m. The addition was such that on day t0 (May 21, 2009) 25 l was added to mesocosm M5 and 50 l each to M1, M3, M4 and M6, while M2 remained unperturbed. On the next day, another 25 l was added to M3, 50 l to M4, 70 l to M6 and 80 l to M1. On the third day, only M1 received another 25 l. Also, on that day, a hole was discovered in the bag of M1, allowing the inflow of surrounding seawater. M1 was therefore excluded from the experiment. The CO_2_ enrichment changed the fugacity of carbon dioxide (fCO_2_) in seawater to about 1,265 ± 120 μatm in M6, 1,080 ± 100 μatm in M4, 815 ± 80 μatm in M3 and 600 ± 60 μatm in M5 on day t1, while fCO_2_ in M2 remained close to that in the surrounding seawater at about 370 ± 40  μatm. Due to considerable biological activity, fCO_2_ ranged from about 675 ± 65,  605 ± 60,  520 ± 50,  420 ± 40 to 310 ± 30 μatm in mesocosms M6, M4, M3, M5 and M2, respectively, on day t4 (at about 7:00), the day of the diurnal cycle. For carbonate chemistry calculations and uncertainty estimates, see "Carbonate chemistry measurements and calculations" section. Addition of CO_2_-enriched seawater changes the concentrations of dissolved inorganic carbon (DIC) while leaving total alkalinity (TA) constant, and is therefore perfectly simulating ongoing ocean acidification (Schulz et al. 2009; Gattuso et al. 2010).

Similar to previous mesocosm CO_2_ perturbation studies (e.g., Engel et al. 2005; Schulz et al. 2008), a phytoplankton bloom was initiated by adding nitrate and phosphate to the bags on day t0, aiming to increase nitrate concentrations from below detection limit to 10 μmol l^−1^ and phosphate from initial concentrations of about 0.15 to 0.65 μmol l^−1^. Since the exact volumes of the bags were unknown (due to the flexible walls), nutrients were added in two steps. The first addition was based on a conservative estimate, significantly smaller than the theoretical volume of 37.7 m^3^. Measured nutrient concentrations in depth-integrated (0–9.5 m) samples collected with an Integrating Water Sampler, IWS (HYDROBIOS), a couple of hours after the addition revealed that water volume was indeed larger and differed between mesocosms by up to 20 %. Nitrate and phosphate were therefore added a second time, in different amounts to each mesocosm, on the morning of day t2 to achieve the targeted addition of 10 μmol l^−1^ of nitrate and 0.5 μmol l^−1^ of phosphate. However, as nutrients are relatively quickly taken up by marine phytoplankton (1.2–1.6 μmol l^−1^ of nitrate between day t0 and day t1), they are not ideal tracers of mesocosm volume. Therefore, it is possible that slightly different nutrient concentrations were added to each mesocosm.

Measurement of nitrate and phosphate followed the methods described in Hansen and Koroleff (1999).

### Carbonate chemistry measurements and calculations

Carbonate chemistry was calculated from pH and total alkalinity (TA) with the temperature- and salinity-dependent stoichiometric equilibrium constants for carbonic acid determined by Mehrbach et al. (1973) as refitted by Lueker et al. (2000). TA was determined by potentiometric titration as described in Dickson et al. (2003) and (2007) on depth-integrated (0–9.5 m) water samples collected with depth-integrating water samplers (IWS Integrating Water Sampler, HYDROBIOS) in each mesocosm and the surrounding water about every other day. pH profiles were measured by means of a CTD-mounted sensor (see "CTD operation section and "pH correction" section for details).

For calculations of reported fCO_2_ levels on day t1, mean water column (0–9.5 m) pH was derived from respective profiles (for pH correction, see "pH correction" section) on this day and used together with linearly interpolated TA values derived from depth-integrated (0–9.5 m) TA measurements in each mesocosm on days t0 and t2. fCO_2_ levels reported for day t4 were derived analogously. Note that TA changed only marginally during the 14 days of the experiment (10–20 μmol kg^−1^), mainly due to nitrate utilization and evaporation.

**Fig. 1:**
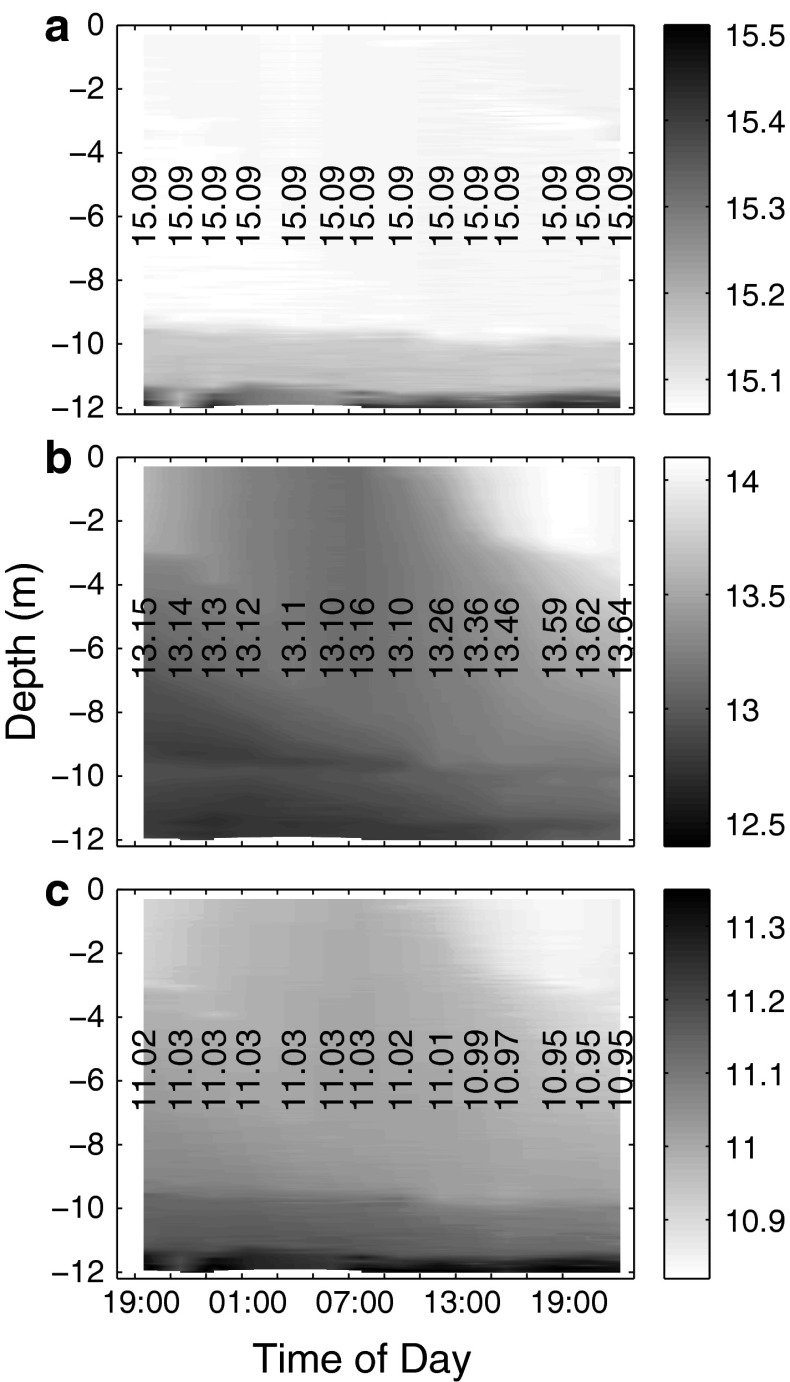
Measured vertical distribution of salinity (**a**), temperature (**b**) and density anomaly σ_T_ (**c**) with time in mesocosm M4. Temperature is reported in degrees Celsius and σ_T_ in kg/m^3^. *Vertical numbers* give mean values of the respective parameter in the water column (0.3–11 m) at each cast. Salinity, temperature and density changes with time are virtually identical in all mesocosms

DIC profiles were calculated from corrected pH profiles (see "pH correction" section for details) and measured depth-integrated (0–9.5 m) TA (on day t5), assuming that total alkalinity was constant with depth. This assumption is supported by the even salinity distribution in the upper 9.5 m of the mesocosms (compare Fig. 1) and negligible contributions of calcifying organisms (D. Rossol, GEOMAR, pers. comm).

Overall uncertainties in calculated fCO_2_ and DIC were determined according to Dickson (2010) from estimated uncertainties in pH (0.04 pH units) and total alkalinity (3 μmol kg^−1^). Uncertainties in calculated fCO_2_ ranged between 40 and 120 μatm, while those in DIC between 9 and 12 μmol kg^−1^. These uncertainty estimates are probably upper limits as coulometric DIC measurements on days t2 and t13 are only 2–5 μmol kg^−1^ off the calculated values. Note that estimated uncertainties are the uncertainties in absolute values/concentrations and that the uncertainties in changes over time (e.g., pH or DIC) relative to starting conditions are less. This is reflected in the relatively smooth increase in pH during the night in all mesocosms, suggesting an uncertainty in pH change of about 0.01, corresponding to an uncertainty in DIC change of about 3 μmol kg^−1^, or even less (for precision of the pH sensor, see next section).

### CTD operation

Profiles of salinity, temperature, turbidity, Chl *a* and pH were collected every two hours starting at 19:00 on day t3 with a cast in the Baltic close to mesocosm M1, followed by M1 (although excluded), M2, M3, M4, M5 and M6. The last CTD cast was on the following day at 22:30 in mesocosm M6. The CTD (memory probe CTD 60M, Sea and Sun Technology) was equipped with an ADM 7-pole conductivity cell (∼0.02 salinity units accuracy and ∼0.005 salinity units precision), a Sea and Sun Technology PT100 temperature sensor (∼0.005 °C accuracy and better than ∼0.001 °C precision), a Seapoint turbidity meter, a Turner Design CYCLOPS-7 fluorometer for Chl *a* and an AMT pH sensor (pressure-balanced glass electrode together with an Ag/AgCl reference electrode in a plastic housing) with a response time of about 1 second (∼0.005 pH units precision). From measured conductivity, temperature and pressure, practical salinity was calculated with the UNESCO PSS-78 formulation proposed by Lewis (1980). Density anomaly (σ_T_) was calculated from salinity, temperature and pressure according to Fofonoff and Millard (1983).

### Chlorophyll *a* correction

Mean water column (0–9.5 m) Chl *a* concentrations, derived from CTD profiles, were compared to measurements from GF/F filters of depth-integrated (0–9.5 m) water samples that were analyzed fluorometrically according to Welschmeyer (1994). A linear regression (adjusted *R* ^2^ = 0.948,   *n* = 65,   *p* ≤ 0.001) was used to correct the CTD-measured profiles for an offset.

### pH correction

For potentiometric pH measurements in seawater, the pH electrode is recommended to be calibrated with synthetic seawater buffers, namely TRIS (2-amino-2-hydroxy-methyl-1,3-propanediol) and 2-aminopyridine adjusted to in situ salinities (Dickson et al. 2007; Dickson 2010). However, this procedure is difficult when the pH electrode is CTD mounted. Therefore, a different approach was chosen here.

On days t2 and t13, depth-integrated (0–9.5 m) water samples were collected for coulometric DIC and potentiometric TA determinations as described in Dickson et al. (2003) and (2007), respectively. From these measurements, pH was calculated on the total scale (pH_T_) with the salinity- and temperature-dependent stoichiometric stability constants for carbonic acid determined by Mehrbach et al. (1973) as refitted by Lueker et al. (2000). Linear regression analyses of calculated pH and mean water column pH, derived from CTD profiles, on day t2 (adjusted *R* ^2^ = 0.986,   *n* = 3,   *p* = 0.054) and day t13 (adjusted *R* ^2^ = 0.987,   *n* = 5,   *p* =  <0.001) were used to correct the CTD-mounted pH sensor measurements and convert them to the total scale.

Potential errors in absolute pH-level determination should not bias the calculated relative changes in pH and DIC in comparison with starting conditions shown here (but also compare "Carbonate chemistry measurements and calculations" section).

### CTD profile visualization

Vertical resolution of measured CTD profiles was typically on the order of about 4–6 cm (casts taken at 0.2–0.3 m/s). Each of the profiles was scaled to a uniform resolution of 2 cm by linear interpolation. Time resolution was on the order of 2 h with a total of 14 casts in each mesocosm and the fjord. Interpolation for 2D contour plots was done with the MATLAB low-level function *contourc* at 100 contour levels.

Profiles shown include measured salinity and temperature, calculated density anomaly, corrected Chl *a* concentrations, corrected pH on the total scale, and corresponding changes in proton concentrations and calculated DIC. Averages of these parameters, representative for 0.3–11 m water depth, were calculated by taking the means of the interpolated profiles. As stated above, estimated uncertainties associated with measured changes in pH and in calculated DIC relative to starting conditions are about 0.01 units and 3 μmol kg^−1^ or less, respectively.

## Results

Temperature, salinity and density characteristics of the enclosed water masses remained relatively constant throughout the measurement period, starting at 19:00 hours on day t3 and ending at 22:30 hours on the following day (compare Fig. 1). There was no detectable change in overall salinity in any of the mesocosms, suggesting that no water was exchanged between the mesocosms and surrounding seawater and, since there was no rain, evaporation was relatively low (less than 1 l per mesocosm). Water temperatures slightly decreased during the night and increased during the day, especially in the upper 3 m which warmed ∼1 °C during day t4.

### Nutrient utilization and biomass build-up

Immediately after the first addition of inorganic nutrients on day t0, the phytoplankton community started taking up the added nitrate and phosphate together with the silicate available. Until the morning of day t3, about half of the added nitrate and a bit less of the phosphate were utilized. On the morning of day t5, measured nitrate concentrations in all mesocosms were at the detection limit of 0.1 μmol l^−1,^ while measured phosphate concentrations were still at about 0.14 μmol l^−1^. This shows that the period of the diurnal cycle, presented here, coincided with the peak of the phytoplankton bloom which was, according to measured Chl *a* concentrations, between the morning of day t4 and day t5 (data not shown).

**Fig. 2:**
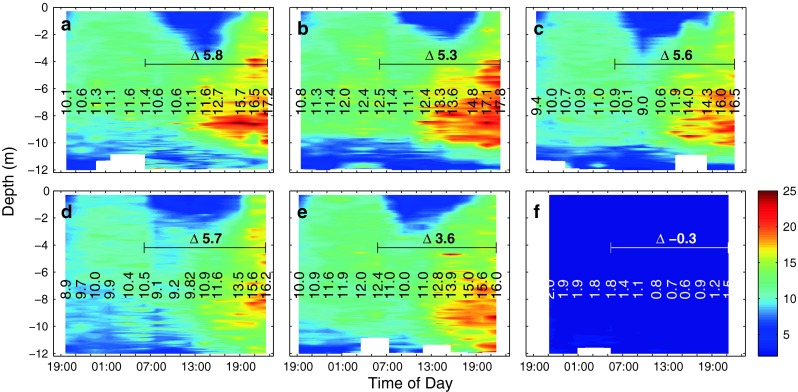
Measured vertical chlorophyll *a* (Chl *a*) distribution and change with time in mesocosms M6 (**a**), M4 (**b**), M3 (**c**), M5 (**d**), M2 (**e**) and the Baltic (**f**) as measured by a CTD-mounted sensor. These values were compared to depth-integrated (0–9.5 m) fluorometric Chl *a* determinations and corrected for an offset (see "Materials and Methods" for details). *Vertical numbers* denote average concentrations (μg l^−1^) between 0.3 and 11 m depth at each cast, while *horizontal numbers* indicate the absolute change in concentrations during the day. Note that the seemingly decreasing concentrations in the upper ∼5 m of the water column starting at dawn until about midday are caused by an attenuation of the chlorophyll fluorescence signal by sunlight and do not reflect actual changes in Chl *a* concentrations (Falkowski and Kolber 1995; Falkowski and Raven 1997)

Promoted by sunny conditions on day t4 and the availability of inorganic nutrients, phytoplankton biomass, measured as Chl *a* concentrations, steadily increased in all mesocosms (Fig. 2). However, the amounts of Chl*a* produced during this day differed between mesocosms, with about 5.8, 5.3, 5.6, 5.7 and 3.6 μg l^−1^ in mesocosms M6, M4, M3, M5 and M2, respectively. Also, Chl *a* concentrations at the end of day t4 differed between mesocosms, ranging from 17.2, 17.8, 16.5, 16.2 to 16.0 μg l^−1^ in mesocosms M6, M4, M3, M5 and M2, respectively. These differences are most likely related to differences in nutrient additions since the volume of the enclosed water differed by up to 20 % between mesocosms, making it difficult to ensure identical nutrient additions. Furthermore, there were also slight differences in the timing of the bloom development. In the surrounding seawater, biomass remained relatively low, fluctuating between ∼0.6 and ∼2.0 μg l^−1^ Chl *a* (compare Fig. 2f).

Microscopic plankton counts showed that the bloom was dominated by diatoms (D. Rossol, IFM-GEOMAR, pers. comm.) with negligible contributions of calcium carbonate producers. The lack of calcification is consistent with the observation that TA changes throughout the experiment could entirely be explained by dissolved inorganic nutrient utilization and evaporation (as measured by increasing salinity).

### Phytoplankton bloom–associated changes in pH and proton concentrations

**Fig. 3:**
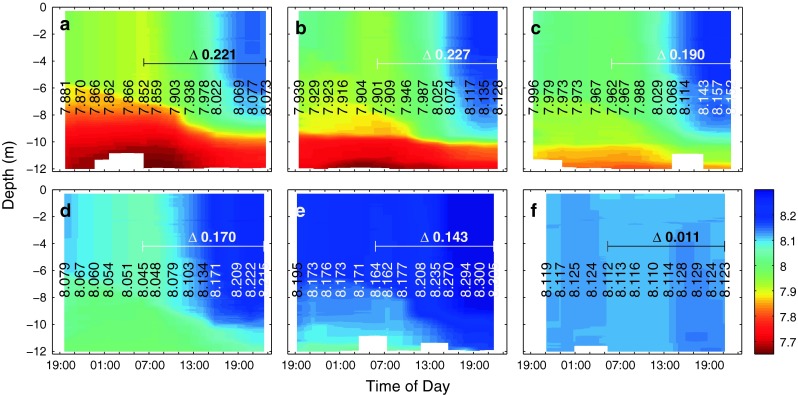
Vertical pH distribution and change with time in mesocosms M6 (**a**), M4 (**b**), M3 (**c**), M5 (**d**), M2 (**e**) and the Baltic (**f**) as measured by a CTD-mounted sensor. The pH is shown on the total scale after correction with calculated pH_T_ from measured total alkalinity and dissolved inorganic carbon (see "Materials and Methods" for details). *Vertical numbers* denote average pH values between 0.3 and 11 m depth at each cast, while *horizontal numbers* indicate the absolute change in pH during the day

**Fig. 4:**
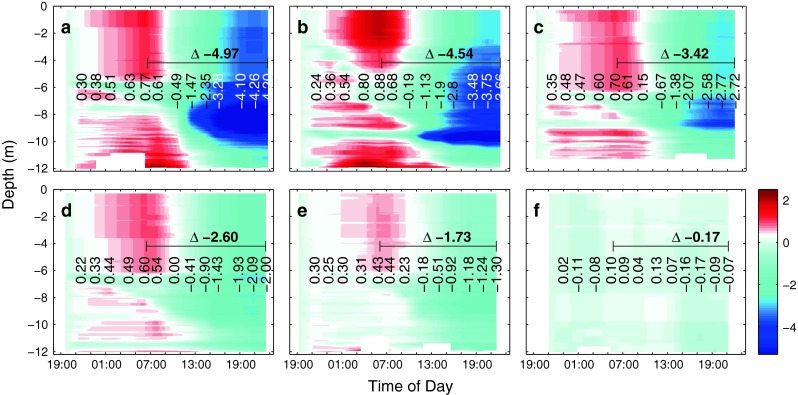
Calculated vertical concentration changes in free hydrogen ions, H_F_^+^ (nmol kg^−1^), relative to starting conditions at about 19:00 in mesocosms M6 (**a**), M4 (**b**), M3 (**c**), M5 (**d**), M2 (**e**) and the Baltic (**f**). Concentration changes were calculated from pH_T_ profiles (compare Fig. 3 and see "Materials and Methods" for details). *Vertical numbers* denote average free hydrogen ion concentration changes in the water column (0.3 and 11 m) relative to starting conditions at each cast. *Horizontal numbers* show the absolute concentration change during the day

Initially, measured pH_T_ levels on day t1 were 7.59, 7.66, 7.78, 7.90 and 8.09 (with an estimated uncertainty of 0.04 units) in mesocosms M6, M4, M3, M5 and M2, respectively, and quite evenly distributed vertically throughout the entire water column (data not shown). Within about three days, following the addition of nutrients, measured pH increased considerably in all mesocosms (compare Fig. 3), caused by the uptake of dissolved inorganic carbon for photosynthetic CO_2_ fixation by phytoplankton. Furthermore, the increase was more pronounced in the upper ∼6 m of the mesocosms in comparison with deeper waters. Diurnal variability was considerable, with pH levels decreasing during the night and increasing during the day. Nevertheless, measured increase during the day was more pronounced than the decrease during the night, meaning that the community was net autotrophic. Averaged over the entire water column, measured pH changes during daytime of t4 were highest at the two lowest pH levels (highest fCO_2_). Toward higher pH in the remaining three mesocosms, the overall change in water column pH was less pronounced and lowest in the mesocosm with the highest pH (lowest fCO_2_) level (compare Fig. 3). Measured variations ranged from 0.227 in mesocosm M4 to 0.143 in M2 (with an estimated uncertainty of less than 0.01 units) and were significantly linearly correlated with CO_2_ level (adjusted *R* ^2^ = 0.9254,   *n* = 5,   *p* = 0.0057). Corresponding changes in [H^+^]_F_ were even more pronounced, being almost three times higher at the highest CO_2_ level compared to the lowest (adjusted *R* ^2^ = 0.9872,   *n* = 5,   *p* = 0.0004) (compare Fig. 4).

### Carbon dioxide gas exchange at the air/sea interface

Measured carbonate chemistry dynamics during the diurnal cycle are driven both by biological activities, such as respiration and photosynthesis, and by physical CO_2_ exchange through the air/sea interface. The impact of air/sea gas exchange on water column DIC inventories, and hence changes in carbonate chemistry speciation, during the diurnal cycle was calculated to be small in comparison with corresponding effects of biological activity, ranging from about 1 to 4 % (see "Appendix"). Note that this corresponds very well with direct gas exchange measurements, made in the mesocosms during another campaign (Czerny et al., in prep.).

### Dissolved inorganic carbon concentration changes by respiration and photosynthesis

**Fig. 5:**
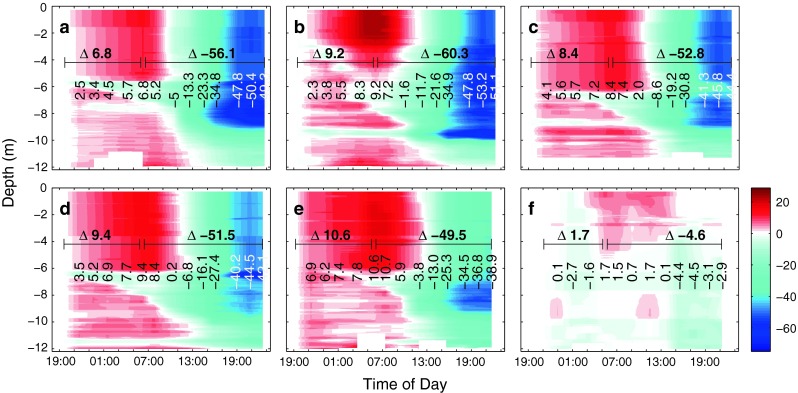
Calculated vertical concentration changes in dissolved inorganic carbon, DIC (μmol kg^−1^), relative to starting conditions at about 19:00 in mesocosms M6 (**a**), M4 (**b**), M3 (**c**), M5 (**d**), M2 (**e**) and the Baltic (**f**). DIC concentrations were calculated from pH_T_ (compare Fig. 3) and measured depth-integrated total alkalinity, TA (see "Materials and Methods" for details). *Vertical numbers* show average DIC concentration (μmol kg^−1^) changes relative to starting conditions in the water column (0.3–11 m) at each cast. *Horizontal numbers* denote concentration changes by net community respiration and photosynthetic carbon fixation during the night and day, respectively

There were significant changes in DIC concentrations during the night and even more pronounced during the day in all mesocosms (Fig. 5). Respiration of the plankton community during the night increased DIC concentrations by 6.6 to 10.9 μmol kg^−1^, while photosynthesis decreased DIC concentrations during the day, ranging from 60.3 in mesocosm M4 to 49.7 μmol kg^−1^ in M2 (with an estimated uncertainty of less than 3 μmol kg^−1^). Differences between mesocosms were partly caused by different biomass standing stocks (compare Fig. 2). When normalizing net community production, corrected for air/sea CO_2_ gas exchange (see "Appendix"), over the entire night/day cycle (26 hours) to Chl *a* concentrations (at 21:00 hours on day t4), there was a statistically significant linear correlation with CO_2_ with 2.744, 2.798, 2.652, 2.612 and 2.501 μmol C/μg Chl *a* per day in mesocosms M6, M4, M3, M5 and M2 at about 675, 605, 520, 420 and 310 μatm fCO_2_, respectively (adjusted *R* ^2^ = 0.8353,   *n* = 5,   *p* = 0.019). This relation was driven by both trends in Chl *a*-normalized community respiration during the night and net community production during the day.

## Discussion

Diurnal variations in carbonate chemistry speciation, such as pH, driven by biological activity are well documented (compare Introduction). The amplitude of these changes is predicted to increase with increasing future CO_2_ levels (decreasing seawater pH) because of declining seawater buffer capacity [compare e.g., Frankignoulle (1994) and Egleston et al. (2010)]. In the following, we will summarize the impact of various biological processes on seawater carbonate chemistry speciation, compare modeled with measured CO_2_-dependent changes in amplitude and discuss potential impacts for marine autotrophs.

The changes in carbonate chemistry speciation observed during the diurnal cycle presented here are direct consequences of two biological processes: photosynthesis and respiration. During photosynthesis, dissolved inorganic carbon (DIC) is consumed. Usually associated are increases in total alkalinity (TA) due to charge-balanced uptake of nitrate (NO_3_^−^) and phosphate (PO_4_^3−^) (Brewer and Goldman 1976; Wolf-Gladrow et al. 2007), although to a much smaller degree. When growth occurs according to the Redfield ratio, NO_3_^−^ and PO_4_^3−^ assimilation generate 0.160 mol of TA for every mole of DIC consumed. Both changes in DIC and TA shift the carbonate system toward higher pH levels and lower carbon dioxide (CO_2_) concentrations. When growth relies entirely on ammonia as nitrogen source, uptake in Redfield proportions reduces TA by 0.142 mol for every mole of DIC consumed. Overall carbonate chemistry speciation changes, however, would be still in the same direction, although slightly smaller in amplitude. Exactly the opposite is happening during respiration (and nutrient remineralization), and its strength in relation to photosynthesis determines whether an ecosystem is net hetero or autotrophic.

### Increased changes in carbonate chemistry diurnal variation in a high CO_2_ world

Despite the fact that photosynthesis and respiration led to similar changes in DIC during daytime, ranging from 49.5 to 60.3 μmol kg^−1^ (compare Figs. 5) in all five CO_2_ treatments of this experiment, diurnal changes in pH were found to be related to actual in situ CO_2_ concentrations, being more pronounced at high than at low levels. Changes in free proton concentrations, [H_F_^+^] were even larger, being almost three times as high at the highest (675 μatm) compared to the lowest (310 μatm) CO_2_ treatment (compare Fig. 4). As stated above, this is the result of lower seawater buffering capacity (Revelle and Suess 1957), meaning that in a high CO_2_ world for the same amount of primary production, respiration or calcification, associated changes in seawater carbonate chemistry speciation such as pH and CO_2_ will be significantly amplified [also compare Frankignoulle (1994) and Egleston et al. (2010)]. Although our observations were made in brackish waters (relatively low salinity and TA), the same applies for more strongly buffered marine waters (higher salinity and TA).

**Fig. 6:**
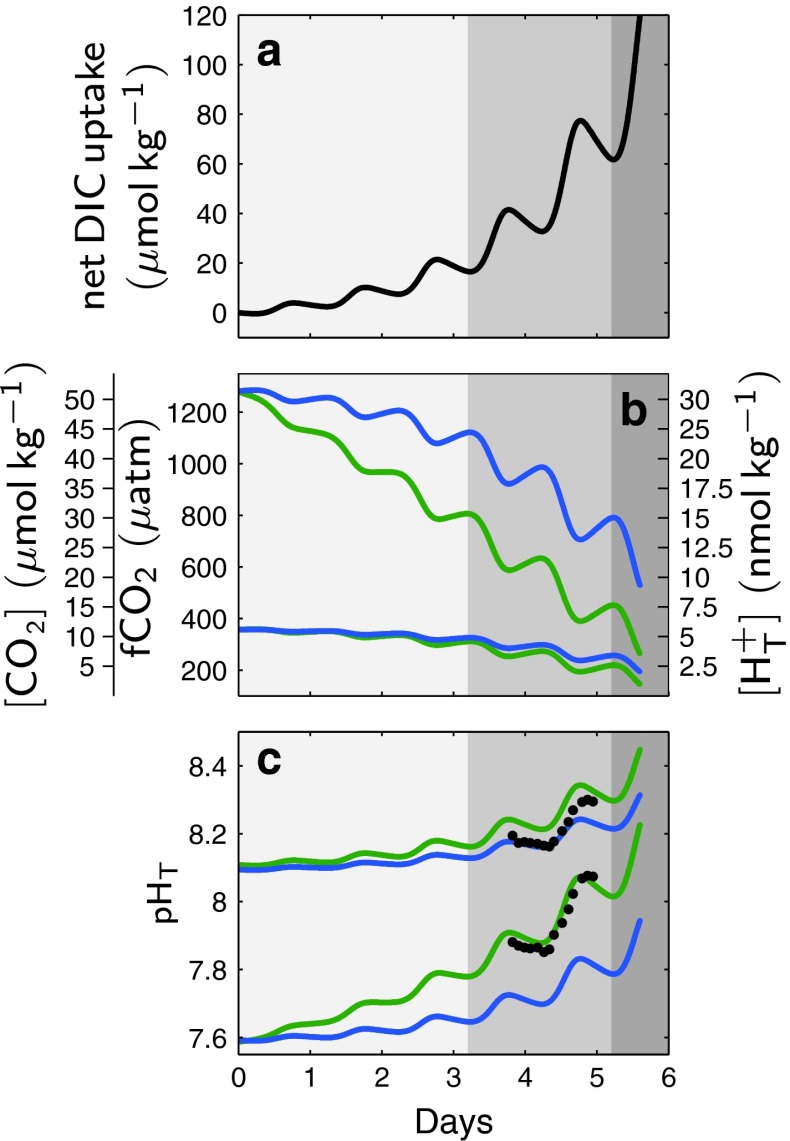
Modeled net community utilization of dissolved inorganic carbon, DIC (**a**) and subsequent changes in proton and carbon dioxide concentration and fugacity, fCO_2_ (**b**), and pH on the total scale (**c**) in the first 5 days following nutrient addition. While *green lines* denote changes at salinity and carbonate chemistry conditions in our coastal setting, the *blue lines* are for open ocean conditions with a salinity and total alkalinity of 33 and 2305 μmol kg^−1^, respectively. *Black dots mark* measured depth-integrated pH values in mesocosm M6 and M2 (compare Fig. 3). The light, intermediate and *dark gray areas* highlight the magnitude of changes expected in waters with increasing nutrient and hence DIC utilization (compare **a**), when moving from oligotrophic open ocean to eutrophic coastal conditions

A simple model of net community production was constructed to visualize several aspects influencing natural carbonate chemistry variability in phytoplankton blooms such as carbon dioxide partial pressure, seawater buffer capacity and nutrient availability (Fig. 6). The model combines light- and biomass-dependent DIC uptake for photosynthetic carbon fixation with biomass-dependent release processes of auto- and heterotrophic respiration (see "Appendix" for details). For reasons of simplicity, it is assumed that both processes are CO_2_ independent (compare Fig. 6a). The model adequately simulates our observations, for instance in pH on day t4 (compare Figs. 3 and 6c). There are small differences between measured and modeled absolute pH levels as overall DIC draw-down was not exactly the same in all mesocosms. Nevertheless, the model clearly demonstrates considerably higher pH and CO_2_ variability at higher seawater CO_2_ levels. There are also differences related to seawater buffer capacity. At the study site in the Baltic Sea, variability in pH and CO_2_ is higher in comparison with open ocean conditions with higher total alkalinity. Furthermore, DIC draw-down driven by nutrient availability clearly shapes carbonate chemistry variability. In this respect, the vast regions of the oligotrophic open ocean can be considered a relatively stable environment.

Considering simultaneously increasing temperatures together with CO_2_ levels, the amplitude of diurnal CO_2_ and [H_F_^+^] variability would be slightly dampened. This is caused by lower CO_2_ solubility at higher temperatures resulting in reduced anthropogenic CO_2_ uptake and therefore diminished reduction in seawater buffer capacity. This effect, however, will be relatively small. For a temperature increase of 3 °C, the decrease in amplitude would be less than 10 % (data not shown).

### CO_2_-dependent carbon fixation

In a previous mesocosm experiment, net photosynthetic carbon uptake was enhanced at elevated seawater CO_2_ concentration (Riebesell et al. 2007). For the same amount of inorganic nutrients taken up, more DIC was drawn down by the plankton community leading to changes in the stoichiometry regarding carbon to nutrient utilization (Bellerby et al. 2008). When normalizing carbon uptake to phytoplankton biomass, a significant trend of higher rates toward elevated CO_2_ is also evident in this study. Chlorophyll *a*-normalized net community DIC uptake, corrected for physical air/sea gas exchange, was about 10 % higher at an fCO_2_ of about 675 μatm compared to 310 μatm. The magnitude of this response is slightly lower than that described previously (Riebesell et al. 2007), yet indicating that this could be a common feature in coastal plankton communities. About half of the overall response was due to changes in nighttime respiration, being reduced at higher CO_2_ levels (compare Figs. 2 and 5). CO_2_-dependent changes in dark respiration have also been described for mono-specific diatom cultures grown at various carbon dioxide concentrations, but there seem to be species-specific differences (Hu and Gao 2008).

It is noted that there are uncertainties associated with our observation of higher Chl *a*-normalized net community production at elevated CO_2_. They are related to our air/sea gas exchange estimate and to uncertainties in changes of measured pH and calculated DIC. The latter should rather increase scatter in the data than produce trends as uncertainties in calculated DIC changes are independent of CO_2_ level. Uncertainties in air/sea gas exchange, however, could cause the observed relationship. Although our estimates compare well with direct measurements of CO_2_ gas exchange during another mesocosm campaign (Czerny et al., in prep), if underestimated by a factor of two, there would be no significant effect of CO_2_ on Chl *a*-normalized net community DIC uptake.

In any case, the phenomenon of enhanced DIC consumption by marine plankton communities would further add to enhanced amplitudes in carbonate chemistry speciation changes at elevated CO_2_. An increase in DIC utilization by 10–20 % would increase the amplitude in carbonate chemistry speciation changes by a similar magnitude.

### Potential implications for marine phytoplankton

Increasing concentrations of CO_2_ and H_F_^+^, together with amplitude variability, could affect various phytoplankton communities differently. It could be argued that communities adapted to considerable natural variability in today's eutrophic regions will have no problems with increasing absolute values in CO_2_ and H_F_^+^ [compare Joint et al. (2010)]. However, there will also be a substantial increase in CO_2_ and H_F_^+^ variability, considerably increasing upper and lower seasonal and diurnal boundaries for CO_2_ and H_F_^+^ (compare Fig. 6). How phytoplankton species in these regions will react to this combined change is difficult to predict. For instance, while the operation of carbon-concentrating mechanisms (CCMs) could be advantageous to quickly balance carbon demand with supply during diurnal CO_2_ variability, the competitive advantage of concentrating CO_2_ intracellularly could be lost at higher absolute seawater CO_2_ levels.

The response of phytoplankton communities in the oligotrophic open ocean, however, appears more straightforward to forecast. Here, any future CO_2_ and H_F_^+^ levels will be far beyond natural variability of today's ocean (compare Fig. 6). This could favor less sensitive species and impact community composition.

## Figures and Tables

**Fig. 7:**
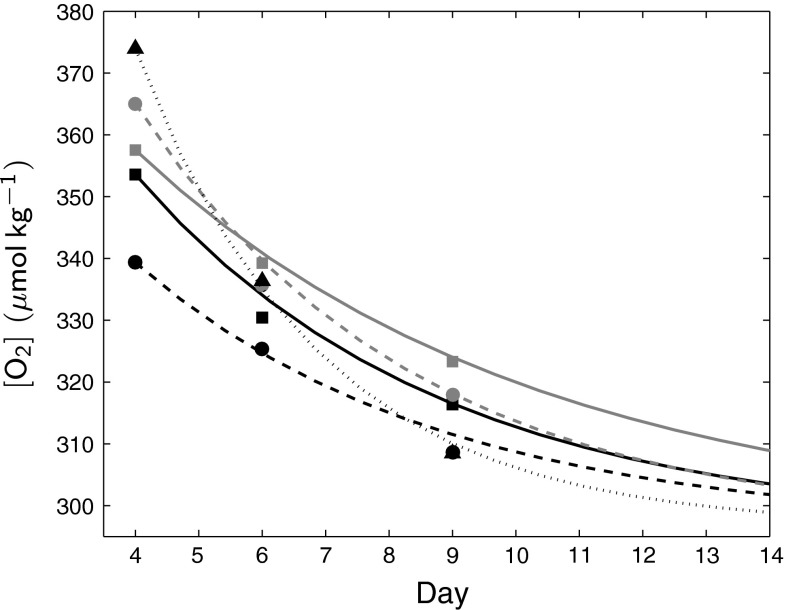
Temporal changes in measured dissolved oxygen concentrations in mesocosms M6 (*black triangles*), M4 (*gray circles*), M3 (*black circles*), M5 (*gray squares*) and M2 (*black squares*) together with fitted concentration changes, leading to phenomenological windspeeds of 6.3, 4.9, 4.6, 3.8 and 4.6 m/s, respectively. See "Appendix" for details

## Appendix

### Air/sea gas exchange calculations

Air/sea gas exchange calculations of CO_2_ and O_2_ in the mesocosms rely on the exact knowledge of the concentration gradient between seawater and overlying air and wind speed which directly scales with the respective flux. Although the wind was blowing during the diurnal cycle (days t3–t4) with an average of about 5.1 m/s (measured a few nautical miles away at 35 m height at the Kiel lighthouse), there was probably almost no wind stress inside the mesocosms because the bags extended to about 1.5 m above the water surface and were covered with a plastic roof further reducing the air exchange [for details, see Riebesell et al., in prep.]. However, air/sea gas exchange is not driven by wind speeds per se but by seawater boundary layer surface texture, modulated by wave action. Since waves were passing through the mesocosms, a windspeed close to 0 m/s for gas exchange calculations would underestimate the actual fluxes. Therefore, we used the decline in measured depth-integrated O_2_ concentrations in the post-bloom phase on days t5, t9 and t11 to derive a phenomenological wind speed. The assumption made here is that changes in seawater oxygen concentrations were dominated by physical gas exchange and biological processes were negligible. This is based on the observation that seawater CO_2_ partial pressures in all mesocosms during this phase were relatively small (data not shown), indicating that photosynthetic carbon dioxide consumption and oxygen evolution were equaled by respiratory carbon dioxide release and oxygen consumption. In essence, the wind speed for calculating the transfer velocity for oxygen ($k_{\text{O}_2}$ given in cm/h) as described in Wanninkhof (1992) was adjusted to fit the measured changes in seawater oxygen concentrations $(\frac{{\rm d}[{\rm O}_2]}{\text{d}t})$ with

1 $$ k_{O_2}= [2.5(0.5246 +1.6256\times 10^{-2}\,tc +4.9946\times 10^{-4}\,tc^{\rm 2})\rm +0.3\,u^2]\rm (Sc/660)^{-1/2} $$

and

2 $$ \frac{{\rm d}[{\rm O}_2]}{{\rm d}t}= k_{O_2} ([{\rm O}_2]_{{\rm sat}}-[{\rm O}_2]_{{\rm meas}}) {A}/(V \rho) $$

with *tc* and *u* being the in situ temperature in degrees Celsius and the wind speed in m/s, respectively, Sc the temperature- and salinity-dependent Schmidt number given in Wanninkhof (1992), and $[{\rm O}_2]_{\rm sat}$ the temperature- and salinity-dependent oxygen saturation in mol/cm^3^ as calculated according to García and Gordon (1992), [*O*_2_]_meas_ the measured depth-integrated oxygen concentrations in mol/cm^3^, *A* the surface area for gas exchange in the mesocosms (31415 cm^2^), *V* the seawater volume estimated to be 30,000  l, ρ the temperature- and salinity-dependent seawater density in kg/l given by Millero and Poisson (1981) and *t* the time in hours. The differential equation 2 was solved numerically with the MATLAB ’ode45’ solver (Shampine and Reichelt 1997), by adjusting *u* to best fit the oxygen changes in the mesocosms during the period from day t4 to day t9. The resulting phenomenological wind speeds were ranging between 3.8 and 6.3 m/s (compare Fig. 7), considerably lower than measured average wind speeds at 35 m height at the Kiel lighthouse of 8.3 m/s. Adopting the median of 4.6 m/s for the CO_2_ flux calculations between days t3 and t4 (measured windspeed of 5.1 m/s) could therefore be considered a conservative approach. DIC concentration changes during the fourteen measurements of the diurnal cycle due to air/sea gas exchange were calculated as

3 $$ \sum^{14}_{i=1} = k_{{\rm CO}_2} {\rm K}_0 (\hbox{fCO}_{2\, \text{air}}- {\rm fCO}_{2\,\text{sea}_{\rm i}})\,{A}/(V \rho) t $$

and

4 $$ {k_{\text{CO}_2}}=[2.5(0.5246 +1.6256\times 10^{-2}\,tc +4.9946\times 10^{-4}\,tc^{2})+0.3\,u^{2}] (\text{Sc}/660)^{-1/2} $$

with ${k_{{\rm CO}_{2}}}$ being the transfer velocity (cm/h) for CO_2_ calculated according to Wanninkhof (1992) with *tc* set to 13 °C and *u* to 4.6 m/s, and K_0_ the solubility of CO_2_ in seawater (Weiss 1974) given in mol kg^−1^ atm^−1^, *fCO*_2 air_ and ${\rm fCO}_{2\,\text{sea}_{\rm i}}$ the carbon dioxide fugacity in air and seawater, respectively, at measurement number *i*, and *t* the time between two consecutive measurements (2 h).

The calculated contribution of air/sea gas exchange to diurnal variations in DIC concentration was 2.1, 1.3, 0.65, −0.23 and −1.2 μmol kg^−1^ in mesocosms M6, M4, M3, M5 and M2, respectively.

### Model parameterization

The model is one of the simplest representations of light- and biomass-dependent DIC utilization, and biomass-dependent release by auto- and heterotrophic respiration as

5 $$ \frac{\text{d }{\rm C}}{\text{d}{\rm t}}= {\rm k\,sin}(\pi t)^4 \text{C} -g \text{C} $$

with C denoting carbon biomass (μmol kg^−1^) and *t* time (d), k representing maximum community gross carbon fixation rate at maximum light level (d^−1^), g comprising all carbon-release processes (d^−1^) and sin(π*t*)^4^ simulating natural diurnal light availability. The model was initialized with measured values for total alkalinity and pH (from mesocosm M6 and M2), initial autotrophic carbon biomass (4 μmol kg^−1^), and k and g (3.15 and 0.6, respectively) to best fit our observations starting at the end of day t3 to the end of day t4 (compare Fig. 3). Carbonate chemistry speciation was calculated as described in the "Carbonate chemistry measurements and calculations" section from TA and DIC, corrected for CO_2_ gas exchange (see above). The differential equation 5 was integrated numerically with the MATLAB *ode45* solver.
